# The Institutional Worker

**Published:** 1921-04-02

**Authors:** 


### THE INSTITUTIONAL WORKER.

*Being a Special Supplement to "The Hospital."*

### INSTITUTION FACTS AND FIGURES.

### Instruction at Welfare Centres.

### Answer to February Question.

The question was :

**Outline a scheme for the collective instruction in hygiene of expectant and nursing mothers, and also of voluntary and salaried workers, at a Maternity and Child Welfare Centre. The expenses of such instruction being provided for by the grant offered for this purpose under the Maternity and Child Welfare Act, 1918.**

### By "Big Ben."

The Wellingborough Infant Welfare Centre is open twice weekly from 2.30 to 4 p.m. The expenses of the Centre are provided by voluntary contributions and a grant from the Ministry of Health. The staff consists of a medical officer and two nurses (all paid) and voluntary workers. One day in the week is the doctor's day, when infants are weighed and the doctor consulted; in alternate weeks expectant mothers are received and advised. On the other day in the week the district nurse attends and talks to the mothers and workers; there is a sewing class and tea. The clothing club in connection with the Centre is open.

Talks to Mothers and Workers.

These last from the beginning of the winter till about Easter. They are held in one large room, and the following are the subjects dealt with :—

Expectant Mothers.

Health; fresh air; exercise; bathing; food, bowels; rest in later months mid-day, with feet raised, no corsets, clothing from shoulders; over-lifting; care of breasts; teeth in early months; preparation of clothes for mother and child; cot for child; drawer, basket, box, if no cot; bed for mother, mackintosh, papers, no spring; room, bright, sunny, with fireplace, windows open; no drains or pipes from closets outside windows.

Nursing Mothers.

*Breast Feeding.*—Nipple and child's mouth kept clean; alternate breasts; regular time of feeding; drink of boiled water between feeds occasionally. *Artificial Feeding.*—Kind of food; cow's milk, sterilised; kind of bottle; hole in teat; where milk is kept; quantity of feed (age); care of bottles not in use. *Importance of Weighing. Sleep.*—Time in pure air. *Bath.*—Temperature, soap, skin, drying, powder. *Habits.*—Regular colour and consistency. *Clothing.*—Weight, warmth, material (wool or flannel), binder, body belt. *Teething.*—Mastication, digestion, cleaning.

At the beginning of the winter a talk is given to the workers alone on ventilation of rooms, attention to fires, drains, closets, teaware, etc.

At the end of the course the mothers had the following questions in mothercraft set to them :—

(1) How do you feed a baby from time of weaning up to twelve months old? Mention each kind of food and times of feeding. (2) How would you dress a baby three months old? Mention each garment and kind of material garment is made of. (3) How would you train a baby to form good habits, from ten days to two years old?

The County Superintendent judged the papers, and only two received under 75 per cent. marks. Three prizes were awarded, and they were distributed at the garden fête held in the summer.

### Ward Inventories.

### Answer to March Question.

The question was :—

**What is the simplest method of making an inventory of linen, crockery, furniture, etc., of wards and Nurses' Home? Give directions and examples, and state whether lists should be printed and hung in the wards and home.**

### By "Interested."

In a hospital where each ward is closed annually for cleaning, the inventory of linen, crockery, and furniture will be an easy matter to make during the time the patients are out, and this arrangement ensures that once a year at least the inventory is accurately made.

Let us suppose, however, that it has to be made during occupation. Dealing with linen first, which is always the most difficult owing to articles being away at the laundry, the laundress should be informed of the approaching stocktaking. She would therefore make special efforts to reduce the linen of that particular ward in her possession to a minimum and be able to say exactly how much is still in the laundry. Similarly the ward sister should arrange to send no articles except absolutely necessary ones, such as draw-sheets, to the laundry for two days before. This should ensure an accurate inventory being made. If possible, it should be done twice yearly. Furniture should also be counted twice yearly.

Crockery has a knack of getting broken or disappearing mysteriously; therefore it is advisable to count it every three months.

Each ward should have a printed list of linen, furniture, and crockery hanging up, so that the sister can satisfy herself from time to time without waiting for the official stocktaking.

As regards the Nurses' Home, it should be possible to count the linen on the day of arrival of the clean articles from the laundry, which presumably is once weekly. Crockery and furniture can be taken as in the wards.

A printed list of articles of crockery and furniture should hang in each dining-room, sitting-room, or bedroom; but in the case of linen it is better for the home sister to have a complete list hanging in her linen cupboard.

### By "Elizabeth."

A simple and efficient method of keeping a hospital inventory is as follows :—

There should be two large books, one for the hospital and one for the nurses' home.

In these books a complete list of the entire effects of the institution should be entered, subdivided into departments, wards, rooms, etc. All articles in each subdivision should be entered under different headings, as fixtures, furniture, bedding, linen, ward utensils, crockery, etc. An annual inventory of the entire property should be taken, and notes made as to losses, renewals, or any other changes.

Each ward or department should be supplied with a smaller book, in which every article appertaining to that department should be entered, the lists corresponding with the large book; and when the inventory is taken the sister should have her book and the secretary or matron the large inventory book, so that any notes may be entered.

It must not, however, be thought that an annual inventory will be sufficient to keep things in order. Far from it; but the annual inventory will be the climax of constant checking of the effects. If each sister has the written list of all things under her charge in tabulated form, and if a systematic method of renewals be arranged, inventory keeping is greatly simplified.

The main furniture is not likely to be lost, but it is necessary to overlook even this from time to time and to refer to the inventory book when there is any difficulty. Linen, crockery, brooms, and brushes are perhaps the articles that are most likely to be lost; every good sister knows the necessity of keeping a constant watch on these goods. Ward utensils, instruments, splints, etc., are very easily lost unless constantly checked.

It is a good plan to have lists in note-books (again corresponding with the lists in the departmental inventory book) of the articles that are most used by the different members of the staff. The staff nurse can be of great help to the sister in sharing the responsibility of the charge of ward utensils and linen; and the wardmaid, if trained to check crockery and other things more immediately under her charge, will take a pride in keeping them correctly.

In the Nurses' Home there should be strict rules to prevent the loss of crockery, cutlery, etc., or the expense of these items will be enormous. Dining-room maids should have lists of the articles under their care, and these should be counted daily. Maids can easily be trained to do this, as they very soon find the necessity for it, but they must be made responsible. It is a good plan to have one day in each week set apart for the renewal of crockery, hardware, etc., the worn or broken article being produced, and the name of the person given in the case of breakages. Another day should be appointed for the renewal of linen, and in this case each article to be condemned must show the mark of its department.

The upkeep of the property therefore depends upon (1) The sister-in-charge knowing exactly for what she is held responsible.

(2) Those working under her being taught to assist by being held responsible for the articles with which they have most to do.

(3) Systematic and regular checking of the several sections of the effects.

(4) Prompt and regular renewals of worn or broken articles, which will prevent other articles being taken for wrong uses.

### ENQUIRIES AND ANSWERS.

### *THE SICK AND IN NEED.*

### Home for Motherless Child.

You could possibly find accommodation for your little motherless girl at the Home for Motherless Children. If you write to the Superintendent, Robert T. Smith, Esq., 25 Warwick Road, Ealing, you will obtain full information of admission. A weekly payment is required, according to the means of the father.

Worried Parent.

### Home or Hospital for Neurasthenic.

The Bethlem Royal Hospital, Lambeth Road, London, S.E., receives voluntary boarders. You might also write to the Medical Superintendents of the following institutions and inquire as to the suitability of the patient in question for admission to their respective institutions: County Mental Hospital, Cane Hill, Coulsdon, Surrey; City of London Mental Hospital, Stone, near Dartford, Kent; and the Ewell Colony, Ewell, Surrey. Give full details of the case when writing.—X. Y. Z.

### Radium for Cancer Patients.

You would possibly obtain the treatment you desire at the Liverpool Hospital for Cancer and Skin Diseases, Myrtle Street, Liverpool. Perhaps your medical adviser would kindly make inquiries for you if he has not already done so. The address of the Radium Institute is 16 Riding House Street, Portland Place, London, W. Both paying and necessitous patients are treated at this institution.—Sufferer.

### Surgical Treatment Required for Clergyman.

There is a special nursing home for clergy, their wives and widows in London. The address is: Hostel of St. Luke, 14 Fitzroy Square, W. Admission is by payment of from 10s. 6d., according to circumstances, or free to necessitous cases. Apply in the first instance to the Secretary.—Curate.

### *EMPLOYMENT AND TRAINING.*

### Training in Cookery.

Apply for particulars of training to the School of Cookery, Buckingham Palace Road, London, S.W. 1, or to the Secretary, King's College for Women, Campden Hill, W. 8.—Tony.

### Post as Stewardess.

You might write to the following steamship companies and inquire if they have any vacancies for stewardesses on board their liners: The Cunard Steamship Co., 30 Cockspur Street, London, S.W.; the White Star Line; Leyland Line; and Atlantic Transport Line. The address of all three lines is 38 Leadenhall Street, London, E.C. 3. You will obtain all the information you require from the respective companies.—K. A. W.

### *MISCELLANEOUS.*

### Repair of Machine Parts, &c.

You could possibly get the damaged parts welded; this would be much less expensive than renewal. Try Messrs. Barimar, Ltd., 10 Poland Street, London, W. 1. The firm undertakes all kinds of metal repair, including the welding of aluminium as well as iron and steel.—R. M. P.

### Book on Medical Electricity.

You will find "Medical Electricity," by H. Lewis Jones, M.A., M.D., F.R.C.P., and revised and edited by L. W. Bathurst, M.D. (Lond.), of great help to you. It is published by H. K. Lewis & Co., Ltd., 136 Gower Street, London, W.C. 1. The Scientific Press, Ltd., 28/29 Southampton Street, Strand, W.C. 2, have recently published an Atlas of the Sensory Cutaneous Nerves by William Ibbotson, M.R.C.S., L.R.C.P., which should be helpful to you. The price is 7s. 9d., post free.—Student.

### Skin Preparation with Iodine Spirit.

The skins of some people, and more especially children, are susceptible to burns from iodine spirit. Great care should therefore be exercised in applying it; there is a tendency sometimes to be far too lavish in the use of the solution. It is said that burns may result from the contact of a Gamgee dressing with skin prepared with iodine, probably due to the formation of iodide of mercury and a resultant caustic action.—T. C. W.

### Maternity Homes.

We would recommend you to apply to the Secretary of the Woman's Mission to Women, 117 Victoria Street, S.W. 1. This Mission has several Maternity Homes for young women expecting a first child, and one for the reception of young women with their infants. The minimum entrance fee to the Maternity Home is £5 10s., and the maternity benefit, which covers maintenance for a period not exceeding fourteen weeks, as well as expenses of confinement. A weekly payment of 12s. 6d. is required for maintenance at the post-Maternity Home. The mother is usually retained with her child for a period of three months, or longer if required.—J. W.

### Hospital Lifts.

An automatic electric lift is undoubtedly a great convenience, and if you can afford the expenditure entailed it would be advantageous in some respects to install one. Do you realise that the cost would be probably not less than £1,200?

If your hydraulic lift is a good one it would perhaps pay you to have it placed in good working order, and await a time for the installation of a new lift when prices are less than they are at present.—G. H.

